# Epilepsia Partialis Continua (EPC) as an Uncommon Initial Presentation of Cerebral Venous Sinus Thrombosis (CVST)

**DOI:** 10.7759/cureus.22394

**Published:** 2022-02-19

**Authors:** Yong Chuan Chee, Teck Cheng Yap

**Affiliations:** 1 Department of Internal Medicine: Neurology, School of Medical Sciences, Hospital Universiti Sains Malaysia, Kota Bharu, MYS; 2 Department of Neurosciences, School of Medical Sciences, Hospital Universiti Sains Malaysia, Kota Bharu, MYS

**Keywords:** focal motor seizure, status epilepticus, venous infarct, epilepsia partialis continua (epc), cerebral venous sinus thrombosis (cvst)

## Abstract

Cerebral venous sinus thrombosis (CVST) is a rare type of cerebrovascular disease that affects mainly young to middle-aged adults. The main clinical presentation of CVST includes progressive headache, focal neurological deficit, disturbance of conscious level, and epileptic seizures, which can occur early or late in the disease process. Generalized seizure has been recognised as the most common seizure subtype among patients with CVST and epileptic seizures. Epilepsia partialis continua (EPC), a subclass of focal motor status epilepticus, has rarely been reported as the initial presenting feature of CVST. Here, an encounter of CVST with an isolated initial presentation of EPC involving the right hand is presented. The initial discordant clinical and radiological findings prompted further investigations that revealed patchy venous infarction along the precentral gyrus that attributed to the occurrence of EPC.

## Introduction

Cerebral venous sinus thrombosis (CVST) is a rare type of cerebrovascular disease that only accounts for 0.5%-1% of all strokes [1]. Epileptic seizures are a common clinical manifestation of CVST, which may occur early or late in the disease process. Approximately 35%-40% of patients with CVST experience epileptic seizures. Major risk factors include supratentorial parenchymal lesions, cortical vein, superior sagittal sinus involvement, and focal motor deficits, with generalized seizure being the more common seizure subtype [1-2]. Epilepsia partialis continua (EPC), a motor subtype of status epilepticus, is often due to abnormalities close to the central sulcus and has varied underlying etiologies. EPC as the initial presenting feature of CVST is exceeding rare. Here, we report a patient with CVST who had an isolated presentation of EPC involving the right hand. Despite the absence of headache, discordant clinical and radiological findings expedited further investigations that revealed venous infarction along the motor precentral gyrus that led to the development of EPC.

## Case presentation

A 52-year-old gentleman with underlying history of chronic smoking and hypertension presented with sudden onset intermittent, prolonged rhythmic jerking involving his right hand persisting more than two days. Each recurring episode involves mainly the fingers and wrist, lasting for at least 30 minutes to 2 hours with spontaneous resolution. No apparent triggering nor relieving factors were identified. The right-handed patient presented fully awake with intermittent rhythmic jerks of the right hand without any further neurological deficit (Video 1). There were no prior thromboembolic events, and family history for neurological and haematological disorders is unrevealing. Physical examination revealed frequent repetitive stereotypical muscle jerks affecting the distal muscle groups of the hand, occasionally radiating up to the forearm. There was no headache, alteration of his conscious state, or impaired cognition. The rest of the neurological examination was normal. Routine blood investigations, including full blood counts, renal function, and liver function tests, were well within the normal range. Key coagulation parameters (PT: 13.6 seconds, aPTT 39.28, INR: 1.00) were also unrevealing. The D-Dimer was however found to be elevated at 3.4 μg/mL(<0.45μg/mL). A clinical diagnosis of epilepsia partialis continua (EPC) was made.

**Video 1:** Rhythmic jerking of the right distal upper limb was persistent with flexion, extension, pronation, and supination of forearm. There was no involvement of facial muscles, left upper limb and bilateral lower limbs.

**Figure 1:**
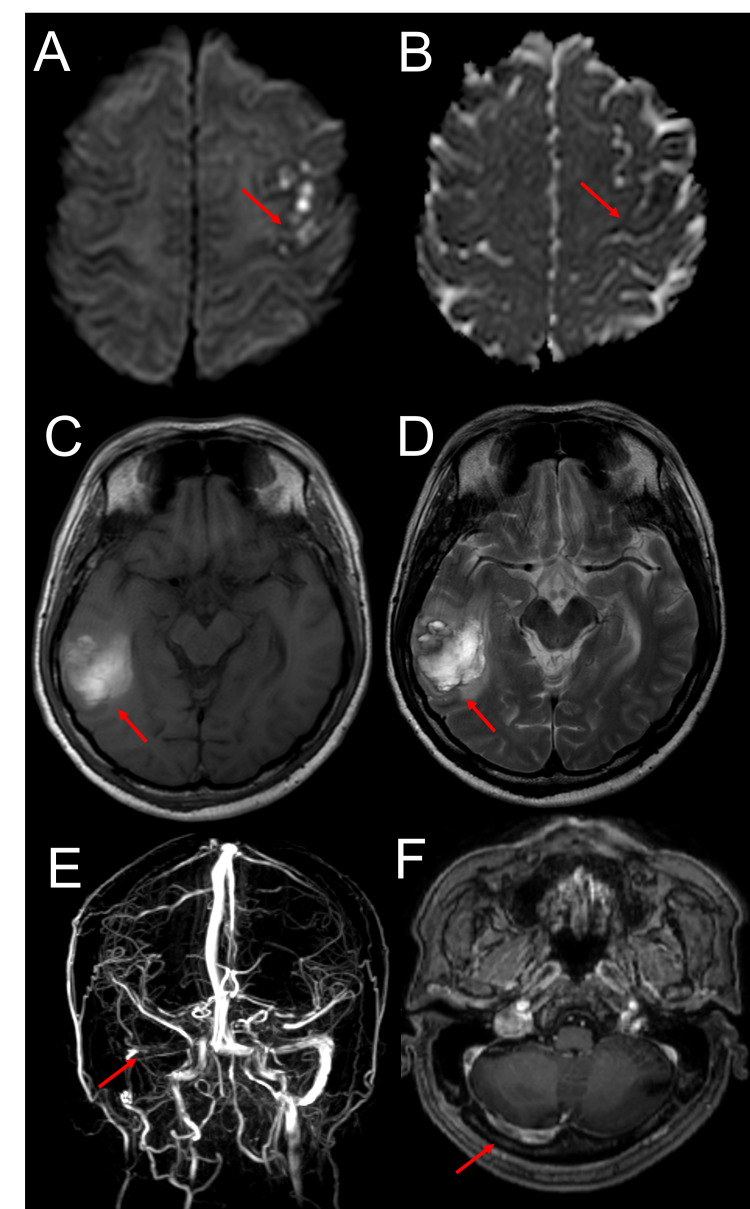
Restricted diffusion is seen at the left precentral gyrus with evidence of high signal on DWI sequence (A) and low signal on ADC (B) suggestive of venous infarction. A well-defined irregular lesion in the right temporal lobe, predominantly hyperintense on T1W (C) and T2W with thin surrounding hypointense rim (D). This is consistent with a combination of vasogenic oedema, cytotoxic oedema, and haemorrhage involving the right temporal lobe consistent with haemorrhagic venous infarction. On MRV, the right transverse and sigmoid sinuses are not seen, as shown by the red arrow (E). Post gadolinium T1 weighted image showing the intraluminal filling defect of the right transverse and sigmoid sinus consistent with dural venous sinus thrombosis (F).

A plain CT (computed tomography) brain scan was done on arrival, and it demonstrated an ill-defined area of hypodensity at the right temporo-occipital region without mass effect. In view of the discordant imaging findings and the clinical presentation, MRI (magnetic resonance imaging) and MRV (magnetic resonance venography) brain was performed to identify suspected lesions along the left precentral gyrus that could best explain the occurrence of EPC of the right hand. MRI revealed long segment thrombosis involving the right transverse, sigmoid sinus, and internal jugular vein. Notably, there was also a linear area of restricted diffusion along the left precentral gyrus that corresponds to the hand area consistent with venous infarction. There was also a combination of vasogenic oedema, cytotoxic oedema, and haemorrhage involving the right temporal lobe consistent with haemorrhagic venous infarct (Figure 1).

The inter-ictal electroencephalography (EEG) recording showed intermittent right temporoparietal theta slowing consistent with focal cortical dysfunction. No epileptiform discharges were seen. The focal motor seizure was aborted with a loading dose of intravenous phenytoin and levetiracetam. The jerking stopped after the completion of the infusion and did not recur. He was also commenced on parenteral anticoagulation with subcutaneous enoxaparin 80mg twice daily (BD) for five days that was subsequently switched to oral Dabigatran 150mg BD. He recovered uneventfully with systemic anticoagulation without treatment-related side effects. Workup for infection, malignancy, autoimmune, and thrombophilia conditions were unrevealing to date.

## Discussion

Epilepsia partialis continua (EPC) is a rare condition where the patient complains of recurrent, occasionally unrelenting focal onset seizure with intact consciousness, which may occur for hours, days, or even years. In the recent International League Against Epilepsy (ILAE) task force report on the classification of status epilepticus, EPC is considered as a subclass of focal motor status [3]. CVST is a rare yet life-threatening condition where recent studies had reported incidence of around 1-1.5/100,000 population/year [4]. Patients with CVST presenting with seizures is not uncommon; approximately around 35%-40% of the patient may experience a seizure at an early or late stage of the disease. However, the occurrence of EPC as the initial presentation of CVST is rarer. Only three cases were reported in a single centred study over 14 years in South India [5].

Any region of the brain cortex can generate focal seizure activity. If sustained or recurrent, this constitutes focal status epilepticus. If the motor cortex is affected, it is termed as EPC, which is described as repetitive rhythmic, unilateral focal motor twitching of limbs and/or face with intact consciousness. EPC has many possible causes comprising both local and systemic aetiologies. The seizures in EPC are believed to be cortical in origin and related to localized encephalitis [6]. Most evidence points to cortical dysfunction being primarily responsible for the clinical manifestation of EPC [7]. The symptoms of motor EPC have also been interpreted as cortical reflex myoclonus [8], but the pathophysiology is probably not the same across the myriad of aetiologies. It is prudent to identify electrophysiological evidence of focal epileptiform activity when making the diagnosis. Although such a causal relationship was not demonstrated on the EEG in this patient, the focal nature of the epileptiform activity and the anatomical correlation with the clinical signs and imaging findings on MRI is indicative of a focal epileptic process.

In this case report, we postulated that the extensive right transverse sinus thrombosis led to venous hypertension and subsequently thrombosis of the cortical vein. Owing to this patchy, venous infarction occurred over a small area along the pre-central gyrus that corresponds to the upper limb somatotropic distribution, resulting in focal cortical irritation and subsequently EPC.

## Conclusions

EPC as the initial manifestation of CVST is an exceedingly rare phenomenon. Recognising EPC as an atypical presentation of CVST would be important, leading to urgent investigation and treatment that yields a reduction in haemorrhagic complications, neurological deficit, and death.
